# The Role of Electrochemotherapy in the Cutaneous and Subcutaneous Metastases From Breast Cancer: Analysis of Predictive Factors to Treatment From an Italian Cohort of Patients

**DOI:** 10.3389/fonc.2021.772144

**Published:** 2021-12-21

**Authors:** Francesco Russano, Paolo Del Fiore, Claudia Di Prata, Andrea Pasqual, Roberto Marconato, Luca Giovanni Campana, Romina Spina, Carlo Maria Gianesini, Alessandra Collodetto, Saveria Tropea, Luigi Dall’Olmo, Sabrina Carraro, Alessandro Parisi, Sara Galuppo, Giovanni Scarzello, Francesca De Terlizzi, Marco Rastrelli, Simone Mocellin

**Affiliations:** ^1^ Soft-Tissue, Peritoneum and Melanoma Surgical Oncology Unit, Veneto Institute of Oncology, IOV-IRCCS, Padua, Italy; ^2^ Department of Surgery, Oncology and Gastroenterology, University of Padua, Padua, Italy; ^3^ Department of Medicine, University of Padua, Padua, Italy; ^4^ Radiotherapy Unit, Veneto Institute of Oncology, IOV-IRCCS, Padua, Italy; ^5^ Scientific & Medical Department, IGEA S.p.A., Carpi, Italy

**Keywords:** electrochemotherapy, breast cancer cutaneous and subcutaneous metastasis, breast Cancer, cutaneous and subcutaneous metastasis, bleomycin, electroporation, breast cancer treatment, therapeutic scenario

## Abstract

The treatment of cutaneous and subcutaneous localizations from breast cancer (BC) is still a therapeutic challenge. Electrochemotherapy (ECT) is one of the available options, and it is characterized by the association between the administration of a chemotherapic agent (Bleomycin) with the temporary raise of permeability of the cellular membrane induced by the local administration of electrical impulses (electroporation). ECT represents an effective therapy for loco-regional control of this disease. This study aimed to investigate the predictive factors of response in cutaneous and subcutaneous localizations from breast cancer treated with ECT. We decided to evaluate the response to this treatment in 55 patients who underwent ECT between January 2013 and March 2020 at our Institute. We performed a monocentric retrospective cohort study. ECT was administered following the ESOPE (European Standard Operative Procedure of Electrochemotherapy) guidelines, a set of criteria updated in 2018 by a panel of European experts on ECT who defined the indications for selecting the patients who can benefit from the ECT treatment and the ones for technically performing the procedure. The responses were evaluated with the RECIST criteria (Response Evaluation Criteria in Solid Tumor). We found after 12 weeks of treatment a complete response (CR) in 64% of our patients. From the analysis divided for subgroups of covariates is emerged that lower BMI, reduced body surface, and absence of previous radiation treatment could be predictive for a better complete response. This study suggests that the efficacy of the ECT treatment is related to the concurrent systemic therapies while administering ECT. The association between ECT and immunotherapy has offered better results than the association between ECT and chemotherapy (p-value = 0.0463). So, ECT is a valuable tool in the treatment of cutaneous and subcutaneous metastases from breast cancer and its efficacy in local control of these lesions improves when it is well planned in a therapeutic scenario.

## Introduction

**Figure 1:**
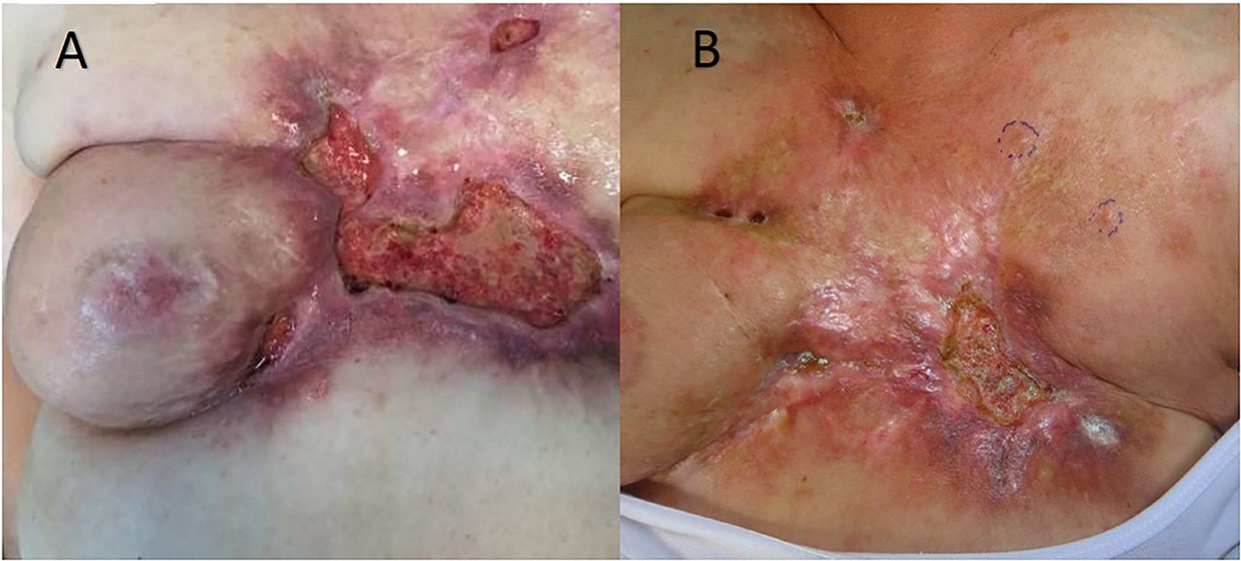
**(A)** Pre-treatment lesions; **(B)** Post-treatment lesions.

BC is one of the most frequent causes of cutaneous and subcutaneous metastases (**Figure 1**). The probability of developing a cutaneous and subcutaneous metastasis from breast cancer varies from 5 to 24% (depending on the stage). Clinically, the lesions are often multiple ones spreading from the scar region to all body. These nodules can ulcerate, bleed, and be very painful. Surgical excision of a single small metastasis is the treatment of choice, while larger lesion or those spreading into a larger area can be very difficult in terms of management. Radiotherapy is a valid option if the area was not already treated with RT, while chemotherapy, immunotherapy, and hormonal therapy can be proposed depending on histology, immunohistochemistry, receptor status, and mutational status of the tumor. Topical application of chemotherapies is only indicated in treating superficial lesions. Electrochemotherapy associates the injection of bleomycin (intravenous or intratumoral) or cisplatinum (only intratumoral) to the local application of electrical impulses that results in an enhanced local drug uptake (1–5). ECT has been proved to be a valid tool in the treatment of cutaneous and subcutaneous metastases (6), demonstrated by a study with a high rate of complete response (73.3%) of the lesions treated with ECT (7). Electrochemotherapy is still seen as a last option for patients with ulcerating, painful, and bleeding lesions, even though there are various studies that can confirm its efficacy (8–17).

We wanted to evaluate the predictive factors to the response to ECT treatment in 55 patients affected by breast cancer cutaneous and subcutaneous metastases, treated at our Institute and to evaluate if ECT treatment could be a valid option for the integrated treatment of breast cancer cutaneous and subcutaneous metastases [in a precise scenario associated with other concurrent therapies: chemotherapy (CT); hormone therapy (HT), immunotherapy (IT), and targeted therapy (TT)].

Our study was conducted on the largest cohort of patients treated with ECT for cutaneous and subcutaneous metastases from breast cancer at our Institute between January 2013 and March 2020. In the case of confluent nodules, this was considered as a single entity and as a single area of treatment.

## Material and Methods

### Study Design

We conducted a retrospective study on the largest cohort of patients (that included 55 patients) treated with ECT for cutaneous and subcutaneous metastases from breast cancer at our Institute between January 2013 and March 2020 The primary objective of this study was to retrospectively evaluate the response to treatment (based on the RECIST criteria). Patients were stratified in two groups based on response to treatment: on one hand the patient who underwent a complete response (CR) and on the other hand the patient who had a partial response (PR), a stable disease (SD) or a progression disease (PD). In case of relapse, we evaluated the site and the time from ECT treatment to the appearing of the new lesions. We evaluated the lesions at different times (the day before the treatment, at 1, 2, 4, 8, and 12 weeks after the treatment and once per month after). We also performed a survival analysis regarding these patients in terms OS (overall survival) after ECT, PFS (local progression-free survival in terms of recurrence of cutaneous and subcutaneous lesions inside the field of treatment), and NLFS (new lesion-free survival in terms of recurrence of cutaneous and subcutaneous lesions outside the field of treatment).

The secondary objective was to evaluate the presence of predictive factors that could influence objective response (OR = CR + PR), both patient- and disease-related.

#### Predictive Factors Influencing Objective Response

•Age of the patient
•Body surface
•BMI
•Site of primary tumor
•Dimension of primary tumor
•TNM
•Clinical stage
•Histotype
•Grading,
•Presence of estrogen receptor and progesterone receptor
•HER2 status in immunohistochemistry (IHC)
•HER2 status *in situ* ibridation (FISH).

### Patients

#### Inclusion Criteria

•Patients with cutaneous and subcutaneous metastases from breast cancer with histological confirmation
•Patients with no indication for surgery
•Dimension of lesion compatible with electrodes used for ECT
•Age >18 y

#### Exclusion Criteria

•Tumor localization near pacemaker or intracardiac device
•Allergy to bleomycin
•Previous treatment with bleomycin with cumulative dose higher than 25,000 UI/m^2^
•Severe kidney failure (serum creatinine >159 umol/L)
•Liver failure
•Pulmonary fibrosis
•Life expectancy less than 3 months
•Epilepsy
•Breast implant in the site of treatment
•Breastfeeding
•Pregnancy

Patients were enrolled in the study regardless of the presence of other distant metastases of the dimensions of the lesions and of the concurrent systemic therapies. All patients gave their consent for data collection and analysis for scientific purposes

### Treatment

ECT is a surgical technique that consists of local applications of electrical impulses at higher voltage that generate a transient electromagnetic field. Electrical pulses (8 pulses of 0.1 ms duration) were delivered using a steering module (Cliniporator, IGEA, Carpi, Italy); this creates a rearrangement of the plasmatic membrane that leads to the formation of transient pores through which the cytotoxic drug can enter the cell. The impulses are conducted through some electrodes created with fine needles in different spatial configuration (exagonal, linear, plate, finger, and single needle) for the surgeon can choose the best configuration for the lesion to treat. Exagonal and linear electrodes can be used for lesion up to 3 cm of depth, while plate and finger electrodes can be used for more superficial lesions. Single needle can be used for bone or visceral lesions. Treatments were conducted following the ESOPE criteria (dose and administration route of bleomycin were decided on the number and the dimension of lesion in the case of intralesional injection and on body surface in the case of intravenous injection). None of the patients had indications for surgery and the only other option left was palliation chemotherapy. Evaluation of efficacy was based on the RECIST criteria.

### Statistical Analysis

Data were analysed by Fisher exact test and Chi square test with a confidence interval of 95%. Survival curves were calculated with Kaplan–Mayer with a confidence interval of 95%. Association between clinically relevant variables and survival was evaluated by Cox regression models and expressed as hazard ratio (HR) with a confidence interval of 95% and compared with the Mantel–Haenszel log-rank test. Due to the low number of patients, we conducted a covariate analysis divided in subgroups (we could not perform a multivariate analysis). We performed a two-sided test and considered statistically relevant a p-value lower than 0.05.

## Results

### Patients

We considered patients who received a diagnosis of BC from March 1982 to June 2017 and subsequently treated with ECT for cutaneous and subcutaneous metastases. We included both patients who received the diagnosis and the treatment at IOV and patients who were treated at other Institutes and then referred to our Center. In that case, some information was missing, and those patients were excluded. Median age at time of ECT administration was 68 y (range 45–92 y), 41 patients (74%) were younger or equal to 75 y, while 14 patients (26%) were older than 75 y. Median BMI was 22 (range 21–26) kg/m^2^, while body surface varied from 1.35 to 2 m^2^ (median 1.75 m^2^).

The characteristics of the tumor were as follows: data on localization (more frequent at upper outer quadrant UOQ and sternal), dimension (more than or equal to 5 cm in 29%, between 2 and 5 cm in 24% and less than or equal to 2 cm in 18% of the cases), T at diagnosis (15% had a T <2 at diagnosis, while 51% had a T more than or equal to 2), nodes involvement at diagnosis (44% had a nodal involvement with N more than or equal to 1), stage at diagnosis (more than II stage in 64% of patients), histotypes (more frequently invasive ductal with 51% of cases and invasive lobular with 13% of cases), ER (positive in 69% of cases), PR (positive in 43% of patients), HER2 status—IHC (+ in 27% of patients, ++ in 22% of patients, +++ in 13% of patients, and negative in 27% of patients), HER2 status—FISH (71% not amplified) and grading (47% with a G2, 47% with a G3 grading) (**Table 1**).

**Table 1:** Characteristic of primary tumors. For each parameter are stated the different variables. For each variable are stated N (number of patients with that variable) and % (percentage).

PARAMETERS	VARIABLES	N	%
**NUMBER OF PATIENTS**		55	100%
**SITE OF PRIMARY TUMOR**	UPPER OUTER QUADRANT	9	15%
	STERNAL	6	11%
	LOWER OUTER QUADRANT	3	5%
	NA	37	67%
	UPPER INNER QUADRANT	9	15%
**DIMENSION**	≤2 CM	10	18%
	2–5 CM	13	24%
	≥5 CM	16	29%
	ND	16	29%
**T**	<2	8	15%
	≥2	28	51%
	ND	19	34%
**N**	0	11	20%
	≥1	24	44%
	ND	20	36%
**STAGE**	<II	5	9%
	≥II	34	62%
	ND	16	29%
**HISTOTYPE**	INVASIVE DUCTAL	28	51%
	INVASIVE LOBULAR	7	13%
	INFLAMMATORY	1	2%
	PAPILLAR	1	2%
	NA	18	33%
**ER**	POSITIVE	38	69%
	NEGATIVE	13	24%
	NA	4	7%
**PR**	POSITIVE	23	42%
	NEGATIVE	26	47%
	NA	6	11%
**HER2 (IHC)**	+	15	27%
	++	12	22%
	+++	7	13%
	NEGATIVE	15	27%
	NA	6	11%
**HER2 (FISH)**	NOT AMPLIFIED	39	71%
	AMPLIFIED	7	13%
	NA	9	16%
**GRADING**	G2	26	47%
	G3	26	47%
	ND	3	5%
**RESPONSE TO TREATMENT**	CR	35	64%
	NR	7	14%
	PR	13	22%
**DUCTAL (50%)**	CR	14	50%
	NR	7	25%
	PR	7	25%
**LOBULAR (13%)**	CR	5	71%
	PR	2	29%
**OTHER (37%)**	CR	16	80%
	NR	2	10%
	PR	2	10%

NA, data not available.

### Treatment

The characteristics of the ECT treatment were the following (**Table 2**): patients were administered the treatment 78 months after the primary breast cancer diagnosis (range 14–441 months). Approximately 16% of the patients had a treatment for a single lesion, 24% for 2 to 5 lesions and 60% for more than 5 lesions. While performing ECT, 27% of patients did not received concurrent therapies, 27% was under chemotherapies, 5% was under immunotherapies, 25% was under hormonal therapies and 14% was treated with multiple therapies combined (2% CT + HT, 5% CT + IT, 2% CT + TT, 5% HT + IT). Approximately 56% of patients who underwent ECT had previously undergone radiotherapy after mastectomy.

**Table 2:** Characteristics of treatment.

N PATIENTS: 55	MEDIAN	MIN	MAX	N (nodules)	N	%
**TIME TO ECT (MONTHS)**	78	14	411			
**NUMBER OF NODULES TREATED**				1	9	16%
				<5	13	24%
				>5	33	60%
**CONCURRENT THERAPIES**			
				LC	15	27%
				CT	15	27%
				IT	3	5%
				HT	14	25%
				CT + HT	1	2%
				CT + IT	3	5%
				CT + TT	1	2%
				IT + HT	3	5%
**PREVIOUS RT TREATMENT**			
				YES	31	56%
				NO	24	44%

N, number of patients, % on total. LC, local control without systemic therapies; CT, chemotherapy; IT, immunotherapy; HT, hormonal therapy; TT, targeted therapy.

We analyzed 55 ECT administered in 55 patients. At 12 weeks from treatment, 64% of the patients showed a complete response (CR) to treatment with progressive disappearance of the lesions verified during subsequent follow-up, 22% of patients had a partial response of the lesions, and 14% stable disease, or a progression disease. During the programmed follow up after the ECT 45% of patients did not experience the rise of new cutaneous nodules, while the others 55% developed new lesions (35% had new lesions outside the field of treatment, while 14% developed new lesions inside the field of treatment). New lesions developed after a time range varying from one to 32 months (median 4 months). The developing of new lesions inside the field of ECT treatment requires less time interval than the developing of new lesions outside of the field of treatment (**Table 3**).

**Table 3:** Recurrence after ECT.

RECURRENCE AFTER ECT						
**RECURRENCE AFTER ECT**					**N**	**%**
	NO				25	45%
	YES				30	55%
**TIME TO RECURRENCE (MONTHS)**		**MEDIAN**	**MIN**	**MAX**		
	TOTAL	4	1	32		
	IN THE FIELD	2	1	21		
	OUT OF THE FIELD	6	1	89		

N, number of patients. % on total.

We also considered the age of the women at the time of ECT, body surface, and BMI. Efficacy of treatment resulted similar in both age classes (more or less than 75 y had a similar CR percentage of respectively 85 and 83%), while lower body surface (less than 1.7 m^2^) and lower BMI (less than 22.5 kg/m^2^) had a higher rate of CR (respectively 87% vs 81% in the case of body surface less than 1.7 and more than 1.7 m^2^ and 90% vs 77% in the case of BMI lower than 22.5 and higher than 22.5 kg/m^2^) (**Table 4**). The response to the treatment was also higher in patients who had a tumor localized in UOQ (CR 88%) or when tumor dimensions were smaller (less than 2 cm: 80% of CR) or greater (more than 5 cm: CR 81%). We did not experience different CR rates in the case of T less or more than 2, but we experienced a different rate of CR in the case of nodal involvement (with N0 the CR was 82%, while with N greater or equal than 1, the CR was 72%), while in the case of lower stage (less than II) CR was of 80% and in the case of higher stage (greater or equal than II), CR was 72%. Histotype with higher response rates was invasive lobular (with CR of 86%). CR rates were also higher in case the primitive tumor was negative for both ER (92%) and PR (85%). Both HER2 (IHC) and grading do not influence response to ECT, while if HER2 is amplified (FISH) we experienced a better CR (86%) **(** **Table 4**). Regarding characteristics of treatment, we considered time to ECT (time interval between diagnosis of primary tumor and ECT administration), number of nodules treated, previous radiotherapy in the field of treatment, and concurrent systemic therapies. We experienced a better CR when ECT is administered in the first 120 months from diagnosis (CR 80% vs 73% if over 120 months), if the number of lesions treated in a single ECT session is more than one (in the case of 2–5 lesions the CR is 92%, while in the case of more than 5 lesions the CR is 85%; in the case of a single lesion the CR is 67%. The response to ECT varied also in the case of previous radiotherapy in the field of ECT treatment. In the case of non-previous RT the CR is 87.5%, while in the case of previous irradiated field the CR is 80% **(** **Table 4**). In the case of concurrent therapies, efficacy of ECT associated to IT is almost 100%, while when ECT is associated to CT the response is lower (CR 67%). We did not evaluate patients who were treated with concurrent multiple systemic therapies (CT + IT, CT + HT, CT + TT, and IT + HT) **(** **Table 4**).

**Table 4:** Response to ECT based on predictive factors referred to primary tumor, demographic predictive factors and predictive factors regarding treatment.

PRIMARY TUMOR CHARACTERISTICS	GROUP	CR %	p-value
**LOCALIZATION**	UOQ	88%	0.5385
	STERNAL	67%	
	LOQ	33%	
**DIMENSIONS**	≤2 cm	80%	0.6554
	2–5 cm	69%	
	≥5 cm	81%	
**T**	T <2	75%	0.910717
	T ≥2	77%	
**N**	N = 0	82%	0.565659
	N ≥1	72%	
**STAGE**	STAGE <II	80%	0.722511
	STAGE ≥II	72%	
**HISTOTIPE**	INVASIVE DUCTAL	50%	0.038
	INVASIVE LOBULAR	71%	
**ER**	POSITIVE	79%	0.4169
	NEGATIVE	92%	
**PR**	POSITIVE	78%	0.7165
	NEGATIVE	85%	
**HER2 (IHC)**	+	73%	0.7021
	++	83%	
	+++	86%	
	NEGATIVE	87%	
**HER2 (FISH)**	NOT AMPLIFIED	79%	10.000
	AMPLIFIED	86%	
**GRADING**	G2	81%	10.000
	G3	85%	
**AGE**	≤75 y	83%	0.807685
	>75 y	85%	
**BODY SURFACE (m²)**	≤1.7 m²	87%	0.572566
	>1.7 m²	81%	
**BMI**	≤22.5	90%	0.202562
	>22.5	77%	
**TIME TO ECT**	≤120 months	80%	0.61209
	>120 months	73%	
**NO. LESIONS TREATED**	1	67%	0.2667
	2	92%	
	>5	85%	
**PREVIOUS RT**	NO	87.5%	0.495556
	SI	80%	
**THERAPEUTIC SCENARIO**	CL	87%	0.0932
	HT	86%	
	CT	64%	
	IT	100%	

CR, complete response.

### Survival

**Figure 2:**
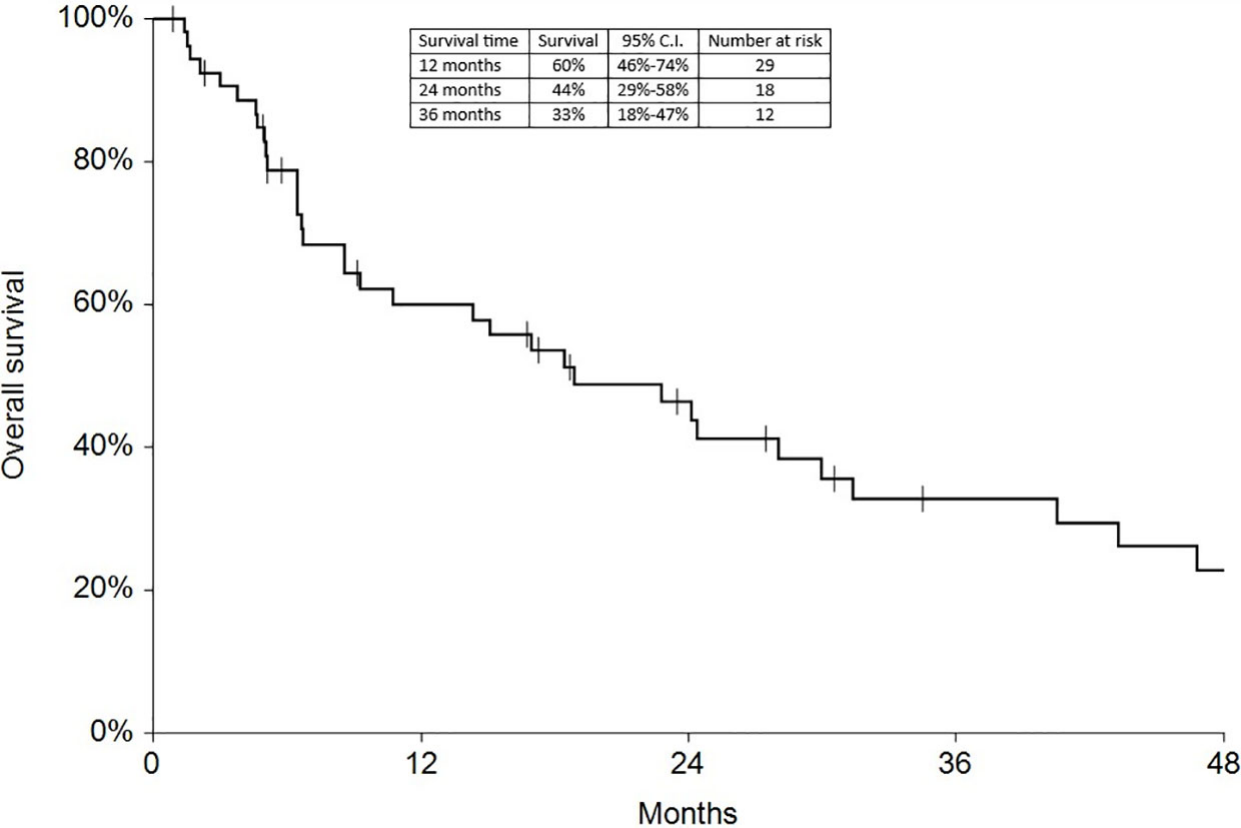
Overall survival at 12, 24, and 36 months.

**Figure 3:**
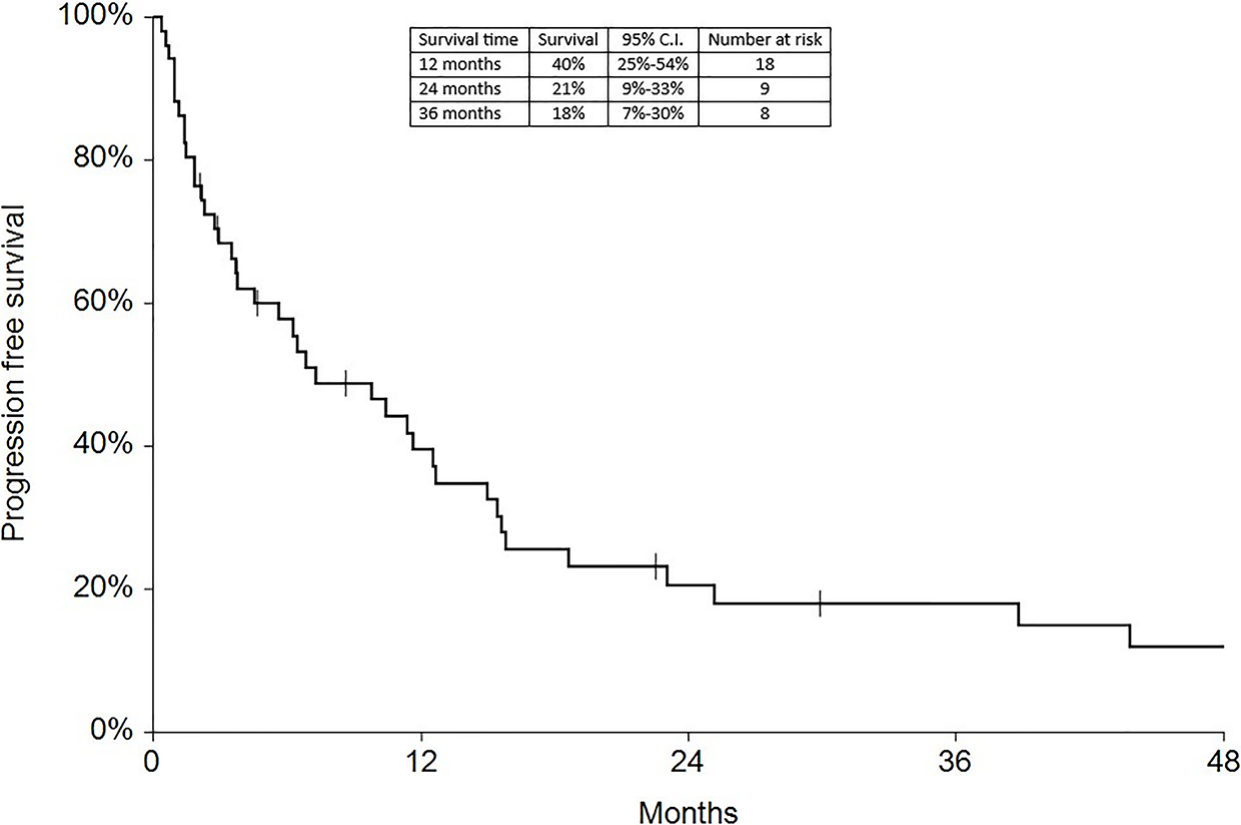
Progression free survival at 12, 24, and 36 months.

**Figure 4:**
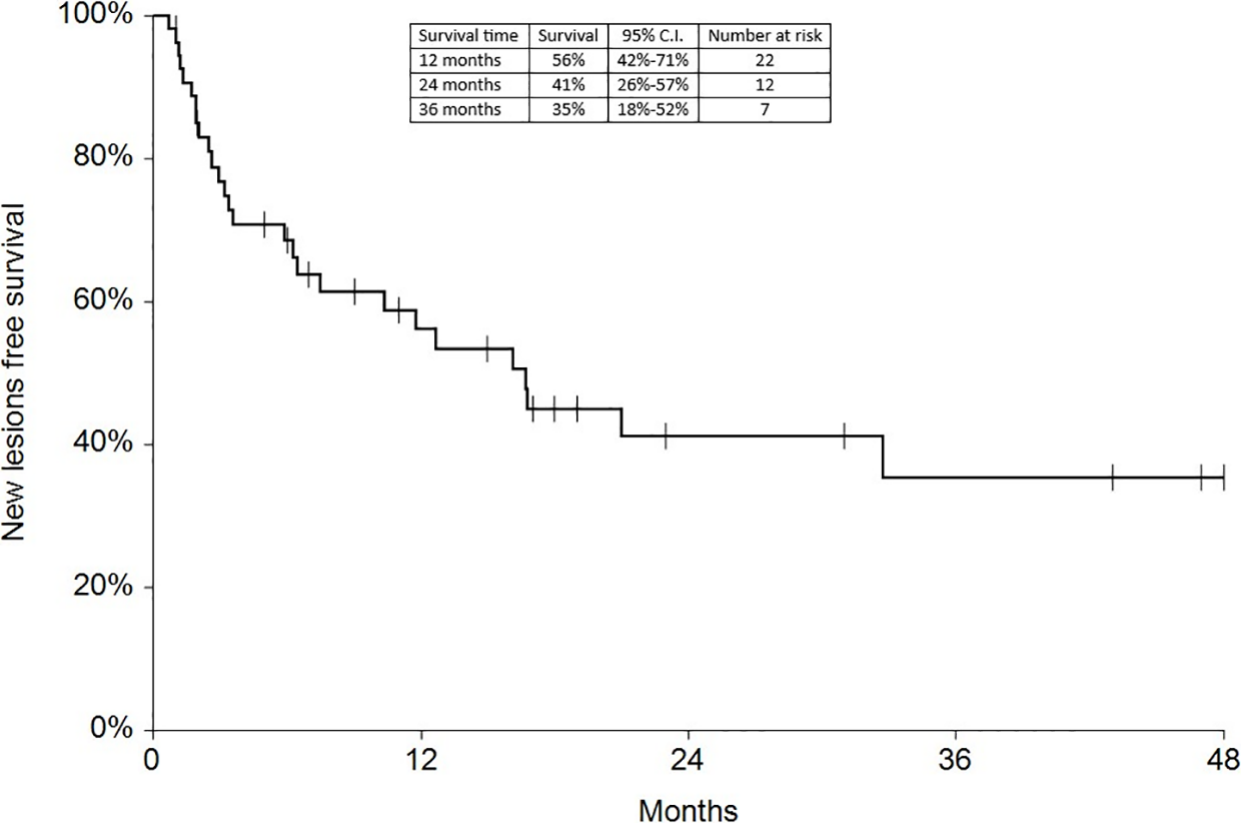
New lesions free survival at 12, 24, and 36 months.

**Figure 5:**
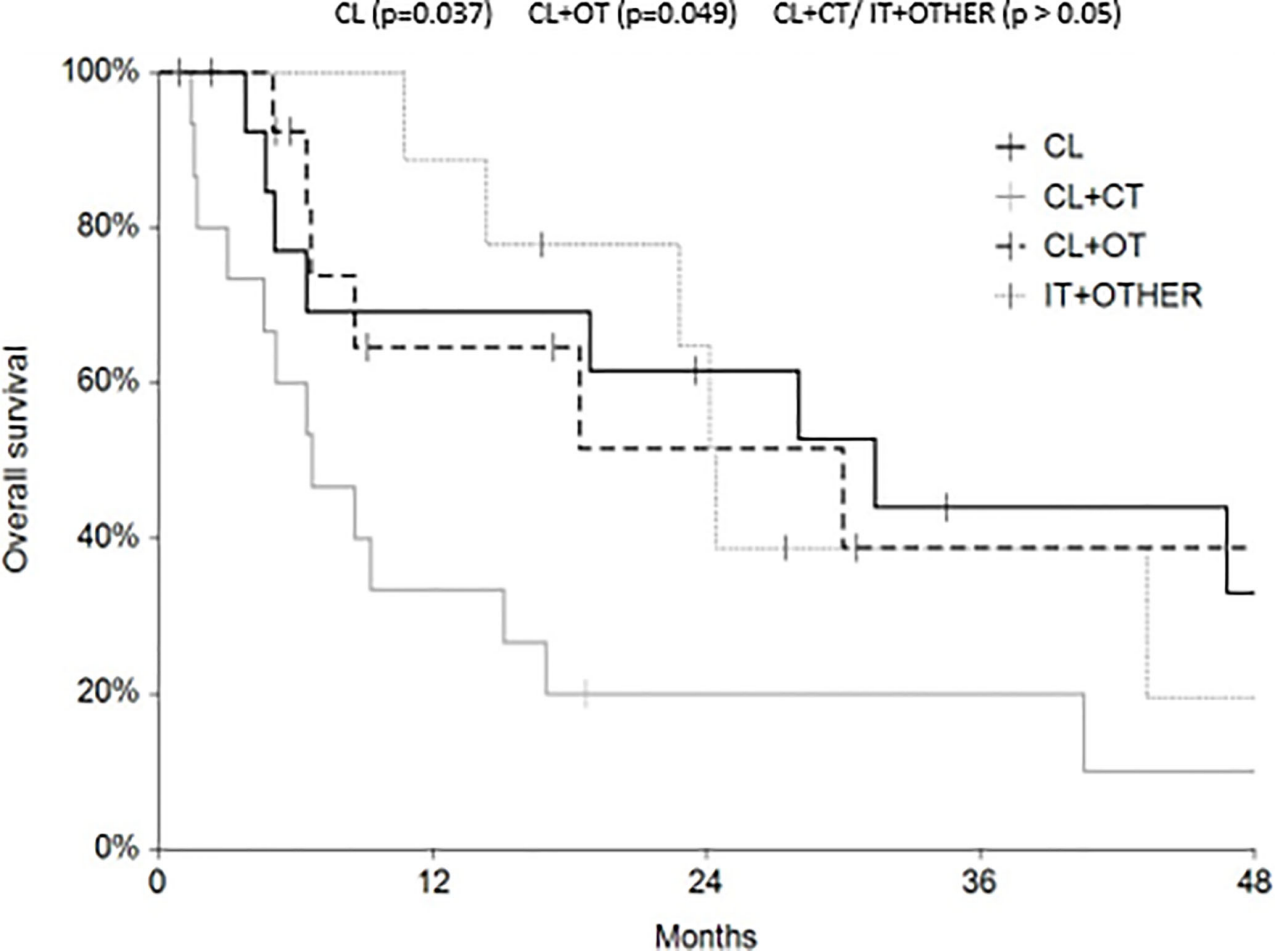
Overall survival (OS) according to the different therapeutic scenario; chemotherapy (CT), immunotherapy (IT), hormonal therapy (HT/OT), and targeted therapy (TT).

**Figure 6:**
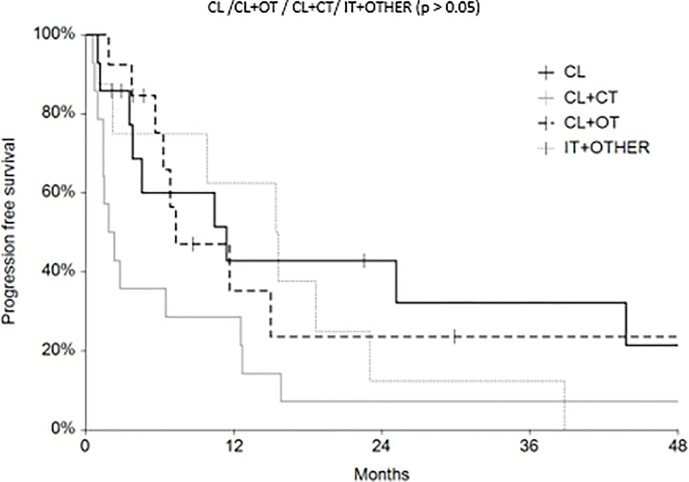
Progression free survival (PFS) according to the different therapeutic scenario; chemotherapy (CT), immunotherapy (IT), hormonal therapy (HT/OT), and targeted therapy (TT).

**Figure 7:**
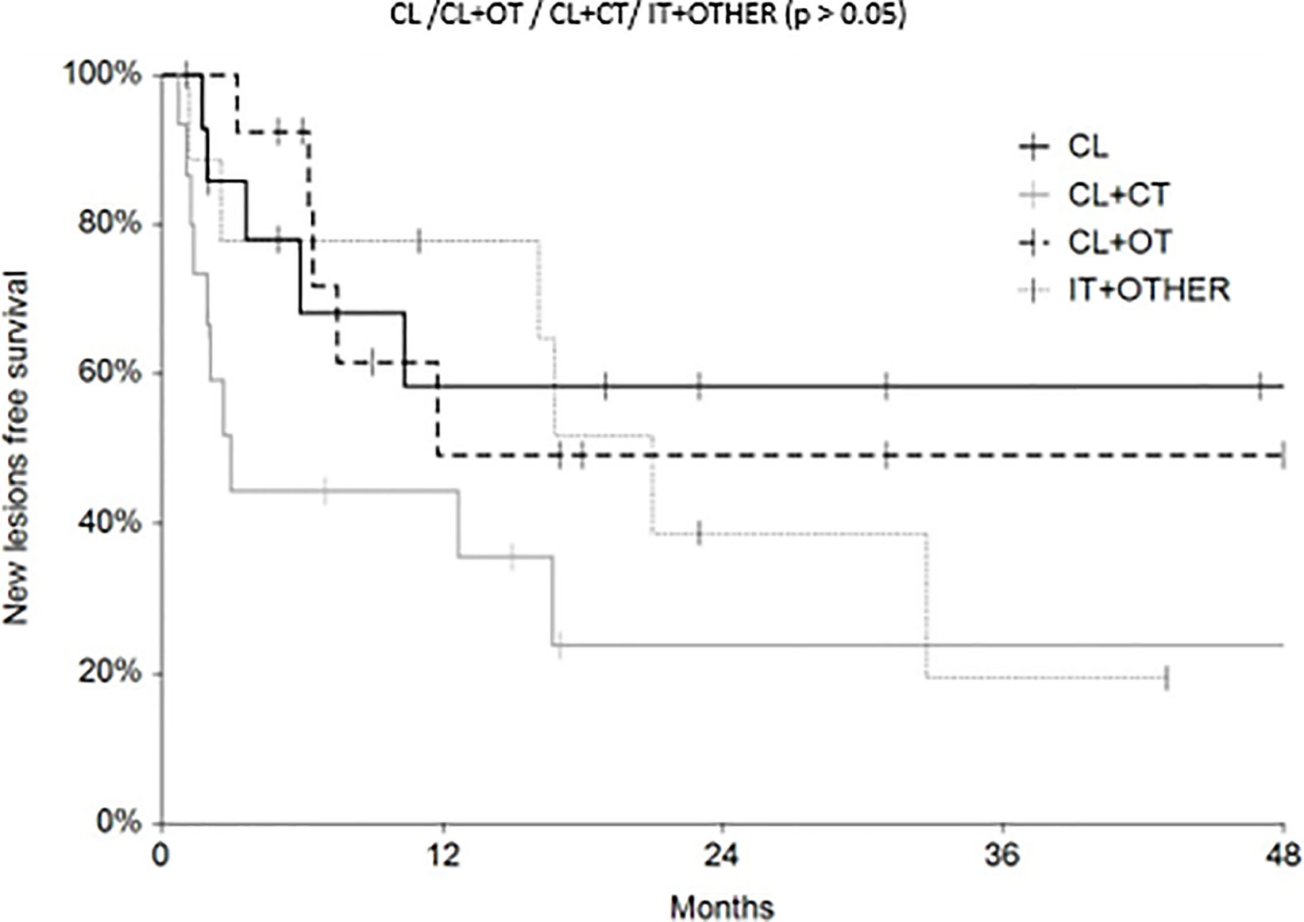
New lesion free survival (NLFS) according to the different therapeutic scenario; chemotherapy (CT), immunotherapy (IT), hormonal therapy (HT/OT), and targeted therapy (TT).

Regarding survival analysis, we evaluated OS (overall survival) after ECT, PFS (local progression-free survival in terms of recurrence of cutaneous and subcutaneous lesions inside the field of treatment), and NLFS (new lesion-free survival in terms of recurrence of cutaneous and subcutaneous lesions outside the field of treatment). We analyzed OS at 12, 24, and 36 months, that resulted respectively with 60, 44, and 33% (**Figure 2**). For PFS at 12, 24, and 36 months the results were the following: 40% of patients at 12 months, 21% of patients at 24 months, and 18% of patients at 36 months did not have recurrence inside the area of treatment (**Figure 3**). In case of recurrence outside the field of treatment (NLFS), at 12, 24, and 36 months we experienced no recurrence in 56, 41, and 35% respectively (**Figure 4**). When we evaluated OS, PFS, and NLFS divided by therapeutic scenarios (concurrent systemic therapies), we discovered that the OS at 24 months is higher in patients who were treated with ECT and concurrent IT, while at 36 months the OS is higher in patients who did not receive concurrent therapies (only local control with ECT). In the overall survival analysis for different therapeutic scenarios, the local control with ECT or local control with ECT plus hormone therapy are superior to the local control with ECT combined with chemotherapy (p = 0.037 and p = 0.049 respectively). All other comparisons among the groups resulted as not significant (p >0.05). The association of ECT with concurrent CT resulted with the worst combination in both short and long term **(** **Figures 5** **–** **7**). For PFS at 24 months, the association with IT still has the highest rates, while at 36 months is the better local control with ECT. Association with CT instead is the therapeutic option with the higher risk of relapse in the short and long term (**Figure 6**). In all these findings a relapse of new lesions outside the field of treatment (NLFS) could also be reported (**Figure 7**). We did not analyze the multiple systemic therapies combination.

**Figure 8:**
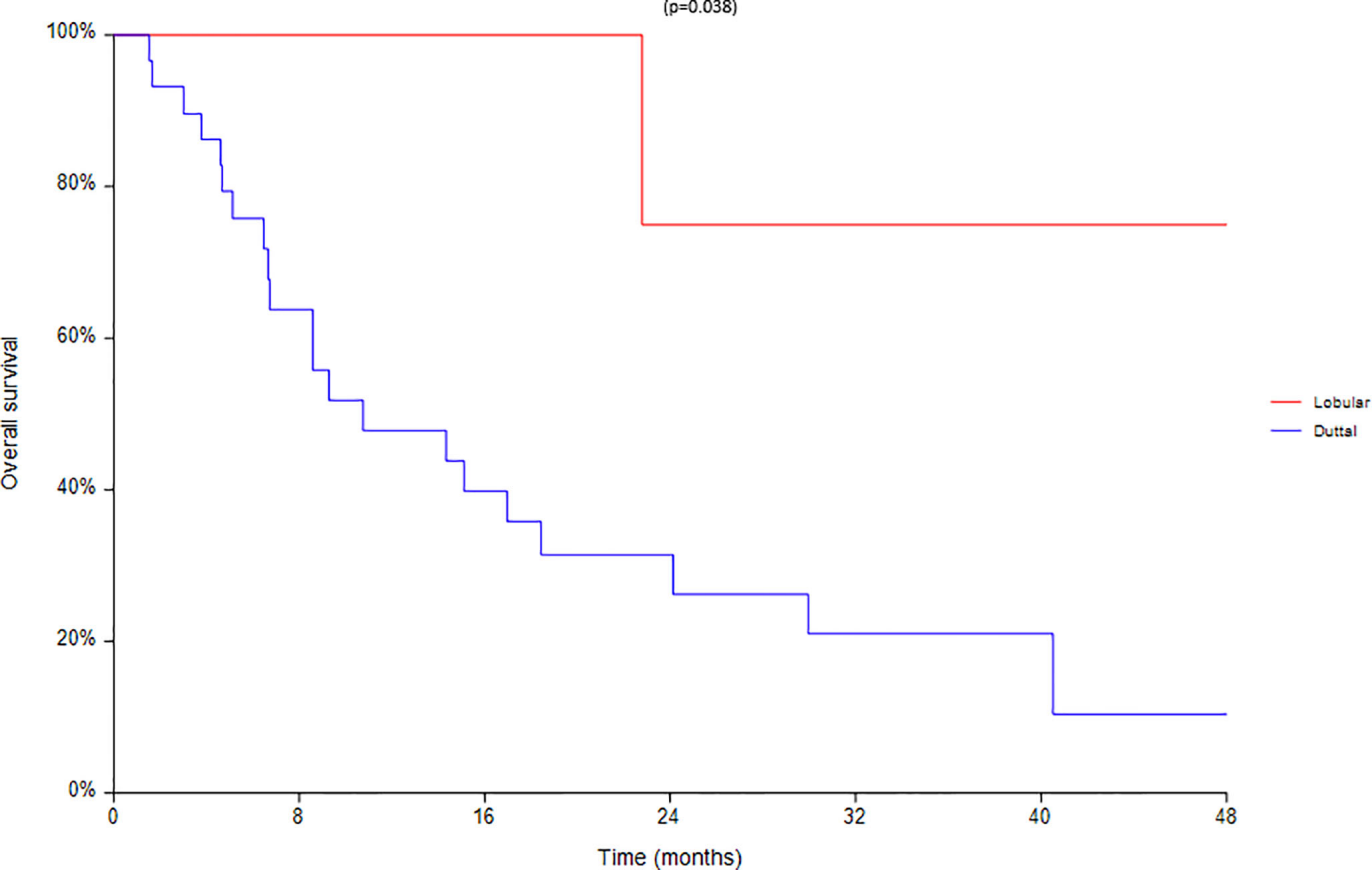
Overall survival according to the different histotypes.

**Figure 9:**
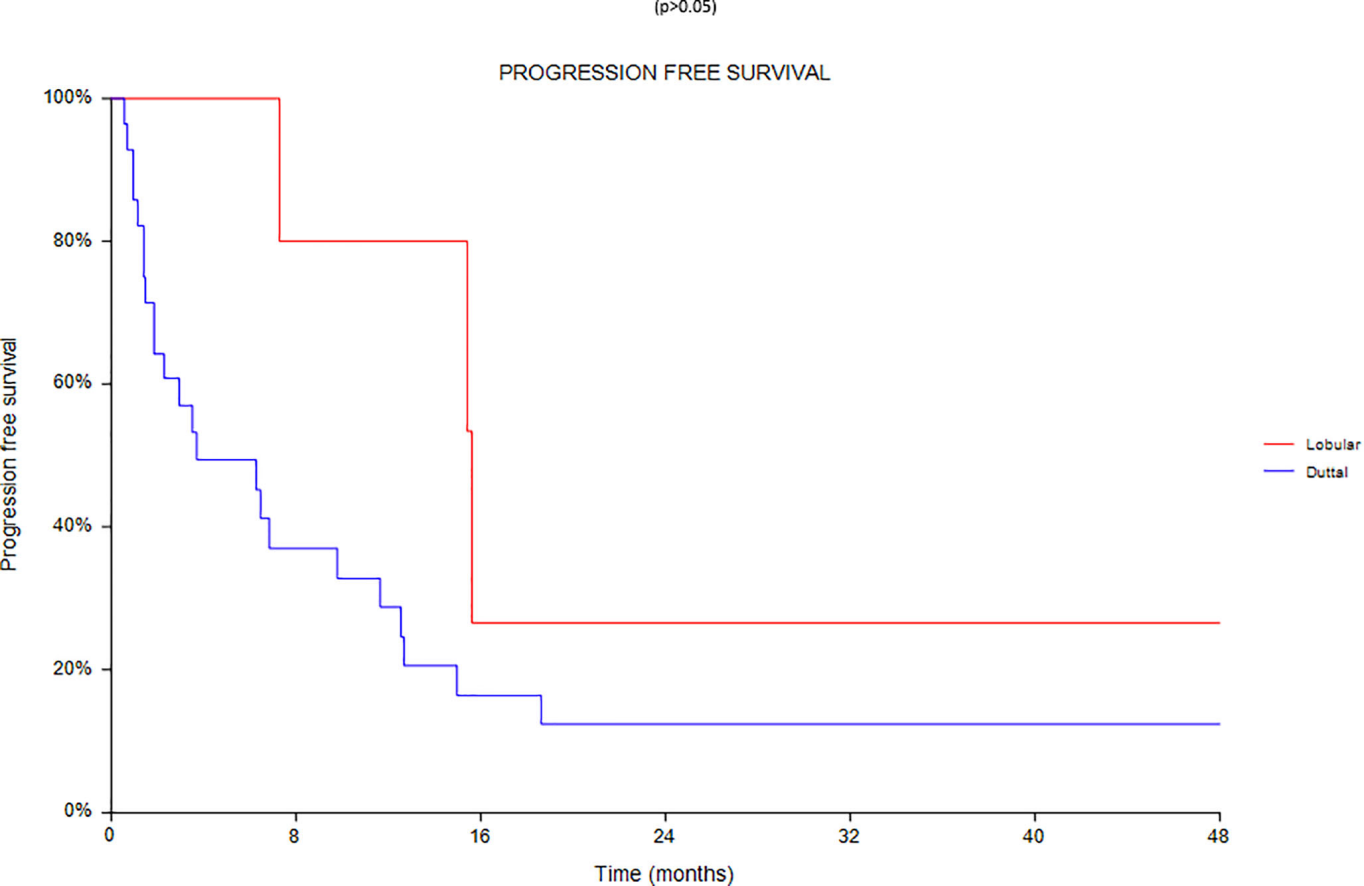
Progression free survival according to the different histotypes.

**Figure 10:**
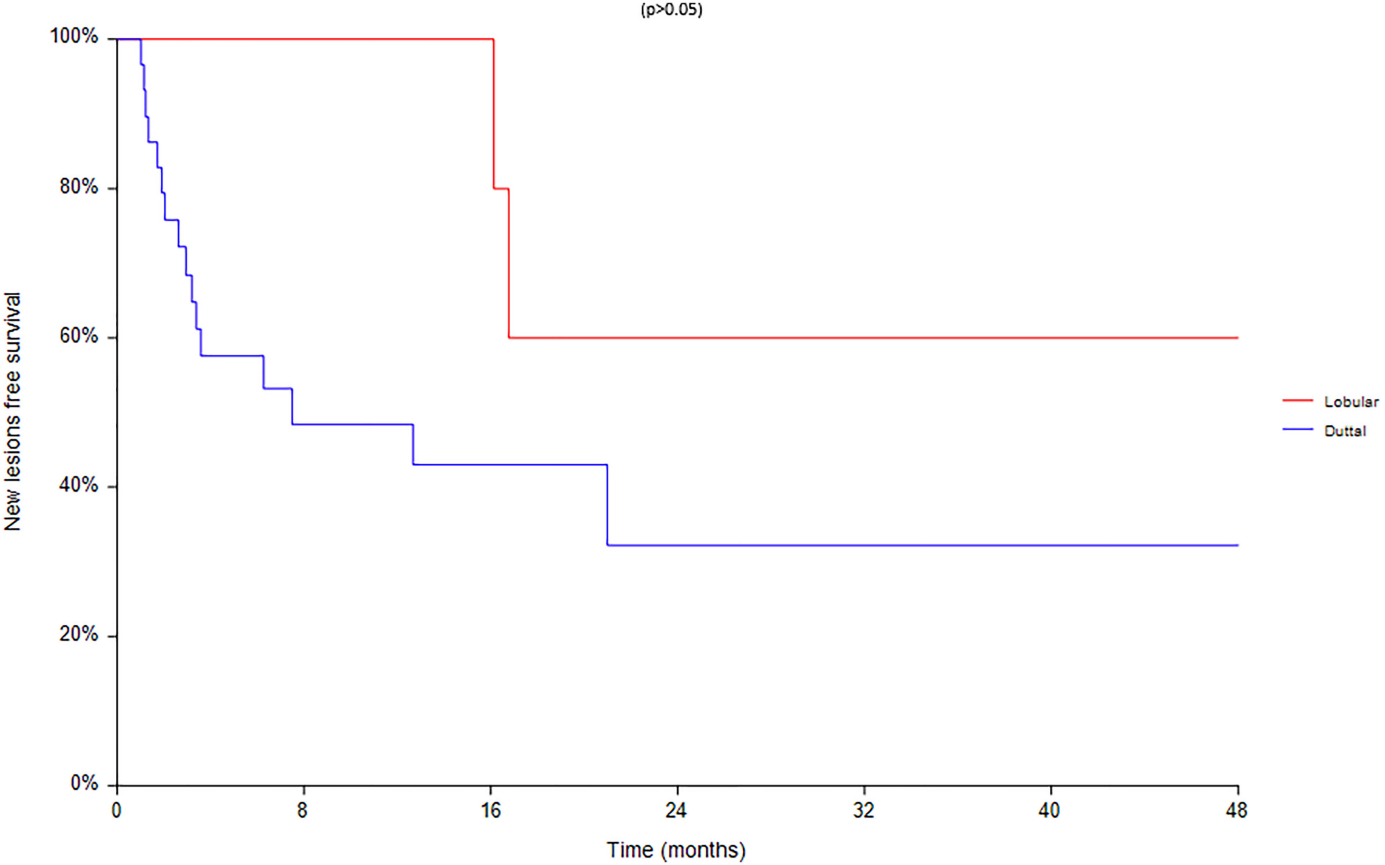
New lesion according to the different histotypes.

Finally an analysis based on subtype was conducted between 28 ductal and 7 lobular carcinomas (**Figures 8**–**10**). We found a significant superiority of lobular carcinoma patients in overall survival (p = 0.038), but not in progression free survival or in new lesions free survival (p >0.05).

## Discussion

Cutaneous and subcutaneous metastases from BC represent a problem in the therapeutic choice; ECT with its objective response rates and low toxicity profile as confirmed by the literature and the clinical experience represents a reliable therapeutic tool.

We confirmed that a patient’s characteristics can influence the rates of complete responses: in our patients, a lower BMI (less than 22.5 kg/m^2^) and a lower body surface (less than 1–77 m^2^) are linked to a higher rate of CR. Obesity can produce an alteration in terms of the drug’s pharmacokinetic (if the drug is administered intravenously), that leads to a different drug’s distribution in the body, so it can lead to a lower drug’s concentration in the site of application of the electrodes. Exceeding fat tissue could also produce hormones that lead to an overproduction/over freeing of estrogen that could increase the hormonal stimulus to tumor lesions. These findings could lead the way to the preference of the intralesional administration of chemotherapic agents instead of the intravenous one in case of higher BMI. In our study we experienced that size and dimension of the primary tumor influence the ECT efficacy that is higher when the primary tumor is in the UOQ and its dimensions are lower than 2 cm or higher than 5 cm. Both characteristics are not already described in literature, so we think that this could be a bias due to our population that consists of a small number of patients, even if this is the larger population described so far. In case of advanced stage, ECT efficacy was lower, probably because advanced stages could be an expression of a more aggressive tumoral behavior, leading to the developing of metastases more resistant to treatment. In our study we did not experience a correlation between positivity to estrogen receptor and higher CR (18). In our case, we found instead the opposite: if there was a positivity to ER, our patients had a lower response. Both negativity to progesterone receptor, and to HER2 were predictive of higher ECT efficacy in our study. We think that it is related to the cellular de-differentiation that is expressed by the negativity to both (PgR and HER2), which could express the cellular inclination to enter in a mitotic phase. The mitotic phase is the moment which the bleomycin could perform its cytotoxic action (bleomycin is indeed a cellular-cycle specific chemotherapic agent). This mechanism could also explain why the metastases derived from a high grading primary tumor (G3) have a better response than those deriving from a lower grade primary tumor (G2). We saw also that HER2 amplification detected by FISH could be a predictive factor of ECT efficacy, because HER2 amplification is linked to the use of trastuzumab, and probably the action of trastuzumab and the cytotoxic effect of bleomycin together could have a synergic effect to these lesions. Regarding the therapeutic issues, we did not gain a statistic value, but we found various trends that could suggest us some patterns. Firstly, we found that there is a minimal difference between the ECT treatment performed before 10 years from the diagnosis of BC and after. It could be because an early relapse after-mastectomy could indicate higher tumor aggressiveness and that makes tumoral cells more sensitive to cytotoxic action of bleomycin. It is also possible that ECT efficacy is higher in younger patients. Secondly, ECT applied on multiple lesions has a higher ratio of complete response than ECT applied on a single lesion. A single lesion has often a higher volume than multiple lesions that often present themselves as a multitude of small nodules. As demonstrated, the smaller dimension of the lesions is predictive of a higher ratio of complete response (15, 16). Thirdly, previous radiation treatment inside the field of ECT administration could negatively affect the efficacy of the treatment, since RT induces a fibrotic reaction onto the tissue, with the creation of a scar tissue that has a different ability to distribute the drugs inside the tumoral lesions, leading to less efficacy of the ECT. It seems instead that ECT applied before the radiation therapy could increase the efficacy of radiation itself (19). We did find with a statistic value that the data regarding the therapeutic scenario in which the ECT is planned could influence its efficacy (20). When ECT is performed in association to IT, the efficacy of the cutaneous lesion’s treatment is almost 100%, while when ECT is performed combined to CT, its efficacy is strongly lowered (**Table 4**). These data are aligned to what is written in the literature, since it has already discussed that the association between ECT and IT seems to induce a bigger activation of the immune system against the tumoral cells (21–28). In our study, the analyses of the survival curves demonstrated that ECT and CT combined is the worst combination, while in the short term the best combination is the ECT with IT. In a long-term setting, the most efficient treatment that resulted is the local control alone (ECT performed without systemic therapies combined). The significant superiority of lobular carcinoma patients in overall survival (p = 0.038), is largely confirmed by literature. We are aware that the long interval of time (from March 1982 to 2017) could somehow limit our study, since some data (immunohistochemistry, for example) in the earliest patients could not be found and since the diagnostic and the therapeutic heterogeneity of this long time could influence our considerations. We think, though, that our study can contribute to validate ECT as a therapeutic option, along with the other traditional procedures, characterized by a high level of efficacy in the treatment of cutaneous and subcutaneous metastases from breast cancer, that are still difficult lesions to treat. Our study confirms the data of the literature regarding the predictive factors of ECT treatment in terms of both primary tumor and treated nodules’ characteristics. We also found with a statistic value that when ECT treatment is planned in a precise therapeutic scenario, its efficacy in the local control of the lesions is strongly influenced.

## Conclusion

Our study confirms the data of the literature regarding the predictive factors of ECT treatment in terms of both primary tumor and treated nodules’ characteristics. We also found with a statistic value that when ECT treatment is planned in a precise therapeutic scenario, its efficacy in the local control of the lesions is strongly influenced. ECT certainly could be a valid option for those patients who have responded incompletely or rather may have a peculiar role in the therapeutic scenarios which all lines of therapy have been concluded.

## Data Availability Statement

The datasets presented in this study can be found in online repositories. The names of the repository/repositories and accession number(s) can be found below: https://zenodo.org/record/5483090#.YTd6mN_OOUk.

## Ethics Statement

According to Italian law (resolution March 1, 2012, Gazzetta Ufficiale n.72 of March 26, 2012), ethics approval and informed consent were not required for the present study owing to its retrospective nature, the use of anonymous data and the fact that it was not associated with any change in patients’ management. All patients gave their written consent to all the diagnostic and therapeutic procedures. The patients/participants provided their written informed consent to participate in this study.

## Author Contributions

Study concepts: PF, FR, and CP, Study design: PF, FR, and CP. Data acquisition: PF, AnP, AIP, RM, LC, SG, AC, and RS. Quality control of data and algorithms: PF and AnP. Data analysis and interpretation: PF, FR, and CP. Statistical analysis: FT. Manuscript preparation: CP and PF. Manuscript editing: CP and PF. Manuscript review: FR, SM, LD’O, MR, GS, and AnP. All authors contributed to the article and approved the submitted version.

## Conflict of Interest

Author FT was employed by company IGEA S.p.A.

The remaining authors declare that the research was conducted in the absence of any commercial or financial relationships that could be construed as a potential conflict of interest.

## Publisher’s Note

All claims expressed in this article are solely those of the authors and do not necessarily represent those of their affiliated organizations, or those of the publisher, the editors and the reviewers. Any product that may be evaluated in this article, or claim that may be made by its manufacturer, is not guaranteed or endorsed by the publisher.

## Abbreviations

ECT, electrochemotherapy; BC, breast cancer; RECIST, Response Evaluation Criteria in Solid Tumor; ESOPE, European Standard Operative Procedure of Electrochemotherapy; BMI, body mass index; CT, chemotherapy; IT, immunotherapy; HT, hormonal therapy; TT, targeted therapy; OS, overall survival; PFS, progression free survival; NLFS, new lesion free survival.
